# Two epidemics, one patient: coinfection by SARS-CoV-2 and influenza in Colombia

**DOI:** 10.15649/cuidarte.4264

**Published:** 2025-12-17

**Authors:** Jhon H. Quintana-Ospina, Luis M. Osorio-Toro, Gustavo A. Urriago-Osorio, Jasbleidy Posu-Barco, Giovanna P. Rivas-Tafurt, Jorge E. Daza-Arana, José M. Oñate-Gutiérrez

**Affiliations:** 1 Specialization in Internal Medicine, Faculty of Health, Universidad Santiago de Cali, Cali, Colombia. Clínica de Occidente Research and Education Group (GIECDO), Clínica de Occidente S.A., Cali, Colombia. E-mail: jhon.quintana00@usc.edu.co Universidad Santiago de Cali Cali Colombia jhon.quintana00@usc.edu.co; 2 Specialization in Internal Medicine, Faculty of Health, Universidad Santiago de Cali, Cali, Colombia. Clínica de Occidente Research and Education Group (GIECDO), Clínica de Occidente S.A., Cali, Colombia. E-mail: luis.osorio01@usc.edu.co Universidad Santiago de Cali Cali Colombia luis.osorio01@usc.edu.co; 3 Specialization in Internal Medicine, Faculty of Health, Universidad Santiago de Cali, Cali, Colombia. Clínica de Occidente Research and Education Group (GIECDO), Clínica de Occidente S.A., Cali, Colombia. E-mail: gustavo.urriago00@usc.edu.co Universidad Santiago de Cali Cali Colombia gustavo.urriago00@usc.edu.co; 4 Clínica de Occidente Research and Education Group (GIECDO), Clínica de Occidente S.A., Cali, Colombia. E-mail: barcojasble@gmail.com Clínica de Occidente S.A. Cali Colombia barcojasble@gmail.com; 5 Specialization in Internal Medicine, Faculty of Health, Universidad Santiago de Cali, Cali, Colombia. Clínica de Occidente Research and Education Group (GIECDO), Clínica de Occidente S.A., Cali, Colombia. E-mail: giovanna.rivas@clinicadeoccidente.com Universidad Santiago de Cali Cali Colombia giovanna.rivas@clinicadeoccidente.com; 6 Physiotherapy Program, Faculty of Health, Universidad Santiago de Cali, Cali, Colombia. Specialization in Internal Medicine, Faculty of Health, Universidad Santiago de Cali, Cali, Colombia. E-mail: jorgedaza.epidemiologia@gmail.com Universidad Santiago de Cali Cali Colombia jorgedaza.epidemiologia@gmail.com; 7 Specialization in Internal Medicine, Faculty of Health, Universidad Santiago de Cali, Cali, Colombia. Clínica de Occidente Research and Education Group (GIECDO), Clínica de Occidente S.A., Cali, Colombia. E-mail: millanonate@gmail.com Universidad Santiago de Cali Cali Colombia millanonate@gmail.com

**Keywords:** COVID-19, Influenza, Coinfection, Aortic Valve Insufficiency, Cardiac Prosthesis Implantation, Mortality, COVID-19, Influenza, Coinfección, Insuficiencia Valvular Aórtica, Implantación de Prótesis Valvular Cardíaca, Mortalidad, COVID-19, Influenza, Coinfecçãos, Insuficiência Valvar Aórtica, Implante de Prótese Valvar Cardíaca, Mortalidade

## Abstract

**Introduction:**

Coinfection by SARS-CoV-2 and influenza (commonly referred to as "Flurona") presents a significant diagnostic and therapeutic challenge in pandemic and postpandemic settings. Although the two viruses share clinical similarities and transmission routes, their treatments differ substantially. The early suspicion of viral coinfection is crucial, particularly in patients with comorbidities or atypical clinical courses. A literature review was conducted in PubMed, Scopus, and Google Scholar (Spanish and English), identifying few documented clinical reports in Colombia.

**Case Description:**

We report the case of an 83-year-old male patient with a significant cardiovascular history, admitted to the intensive care unit for congestive heart failure and severe aortic valve disease. During hospitalization, the patient developed respiratory failure, and coinfection with influenza and SARS-CoV-2 was confirmed Treatment included oseltamivir, oxygen therapy, and therapeutic thoracentesis. Transcatheter aortic valve implantation was indicated, but the patient died during the procedure.

**Conclusion:**

Coinfection by SARS-CoV-2 and influenza should be considered in the differential diagnosis of patients with acute respiratory distress, particularly in contexts of concurrent viral circulation. Prompt recognition enables targeted therapeutic intervention. A multidisciplinary approach is essential to optimize the prognosis in patients with complex comorbidities.

## Resumen

**Introducción:**

La coinfección por SARS-CoV-2 e Influenza (conocida como Flurona) representa un reto diagnóstico y terapéutico relevante en el contexto pandémico y pospandémico, dada la similitud clínica y mecanismo de transmisión entre ambos virus, pero con diferencias importantes en su tratamiento. La sospecha oportuna de coinfección viral es clave, especialmente en pacientes con comorbilidades o evolución clínica atípica. Se realizó una revisión de literatura en PubMed, Scopus y Google Académico (español e inglés), identificando escasos reportes clínicos documentados en Colombia.

**Descripción del caso:**

Paciente masculino de 83 años, con antecedentes cardiovasculares significativos, hospitalizado en unidad de cuidado intensivo por insuficiencia cardíaca congestiva y valvulopatía aórtica severa. Durante la hospitalización desarrolló insuficiencia respiratoria, confirmándose coinfección por Influenza y SARS-CoV-2. Recibió tratamiento con Oseltamivir, oxigenoterapia y toracocentesis terapéutica. Se indicó implantación valvular aórtica transcatéter (TAVI), pero el paciente falleció durante el procedimiento.

**Conclusiones:**

La coinfección por SARS-CoV-2 e Influenza debe ser considerada en el diagnóstico diferencial de pacientes con compromiso respiratorio agudo, particularmente en contextos de circulación viral simultánea. El reconocimiento oportuno permite una intervención terapéutica dirigida. El abordaje multidisciplinario es fundamental para optimizar el pronóstico en pacientes con comorbilidades complejas.

## Resumo

**Introdução:**

A coinfecção por SARS-CoV-2 e Influenza (conhecida como Flurona) representa um desafio diagnóstico e terapêutico significativo no contexto pandêmico e pós-pandêmico, dada a semelhança clínica e o mecanismo de transmissão entre os dois vírus, mas com diferenças importantes em seu tratamento. A suspeita precoce de coinfecção viral é fundamental, especialmente em pacientes com comorbidades ou curso clínico atípico. Uma revisão bibliográfica foi realizada no PubMed, Scopus e Google Acadêmico (espanhol e inglês), identificando poucos relatos clínicos documentados na Colômbia.

**Descrição do Caso:**

Um paciente do sexo masculino, de 83 anos, com histórico cardiovascular significativo, foi hospitalizado na unidade de terapia intensiva por insuficiência cardíaca congestiva e doença valvar aórtica grave. Durante a hospitalização, ele desenvolveu insuficiência respiratória e a coinfecção por influenza e SARS-CoV-2 foi confirmada. O paciente recebeu tratamento com oseltamivir, oxigenoterapia e toracocentese terapêutica. O implante transcateter de válvula aórtica (TAVI) foi indicado, mas o paciente faleceu durante o procedimento.

**Conclusões:**

A coinfecção por SARS-CoV-2 e influenza deve ser considerada no diagnóstico diferencial de pacientes com desconforto respiratório agudo, particularmente em contexto de circulação viral simultânea. O reconhecimento oportuno permite intervenção terapêutica direcionada. Uma abordagem multidisciplinar é essencial para otimizar o prognóstico em pacientes com comorbidades complexas.

## Introduction

Coronavirus disease 2019 (COVID-19) is caused by the severe acute respiratory syndrome coronavirus 2 (SARS-CoV-2), first identified in December 2019 in Wuhan, China. This novel viral entity triggered a global pandemic characterized by high transmissibility, substantial morbidity, and significant mortality, particularly among individuals with risk factors such as advanced age, cardiovascular or respiratory disease, and immunosuppression^1^.

Influenza, by contrast, is a seasonal respiratory infection that typically circulates during the colder months in both hemispheres. Like SARS-CoV-2, it spreads through respiratory droplets and aerosols. Both entities have a clinical presentation characterized by fever, cough, dyspnea, headache, myalgia, and fatigue. Imaging findings such as bilateral pulmonary infiltrates may also overlap, although subtle radiographic differences can aid in the differential diagnosis^2^.

During peaks of COVID-19 transmission, nonpharmacological interventions, including mask use, lockdowns, and hand hygiene, led to a marked reduction in the circulation of other respiratory viruses, including influenza. However, as these control measures have waned, the simultaneous circulation of multiple respiratory pathogens has re-emerged, increasing the likelihood of viral coinfections such as concurrent SARS-CoV-2 and influenza—colloquially referred to as Flurona^3^.

Although considered infrequent, this coinfection has been associated in several studies with increased risks of hospitalization, need for intensive care, and mortality, especially in older adults and patients with cardiovascular comorbidities. This results in a diagnostic and therapeutic challenge, as clinical overlap may delay etiologic diagnosis and the initiation of timely targeted treatment^4^.

## Case description

**Figure 1:**
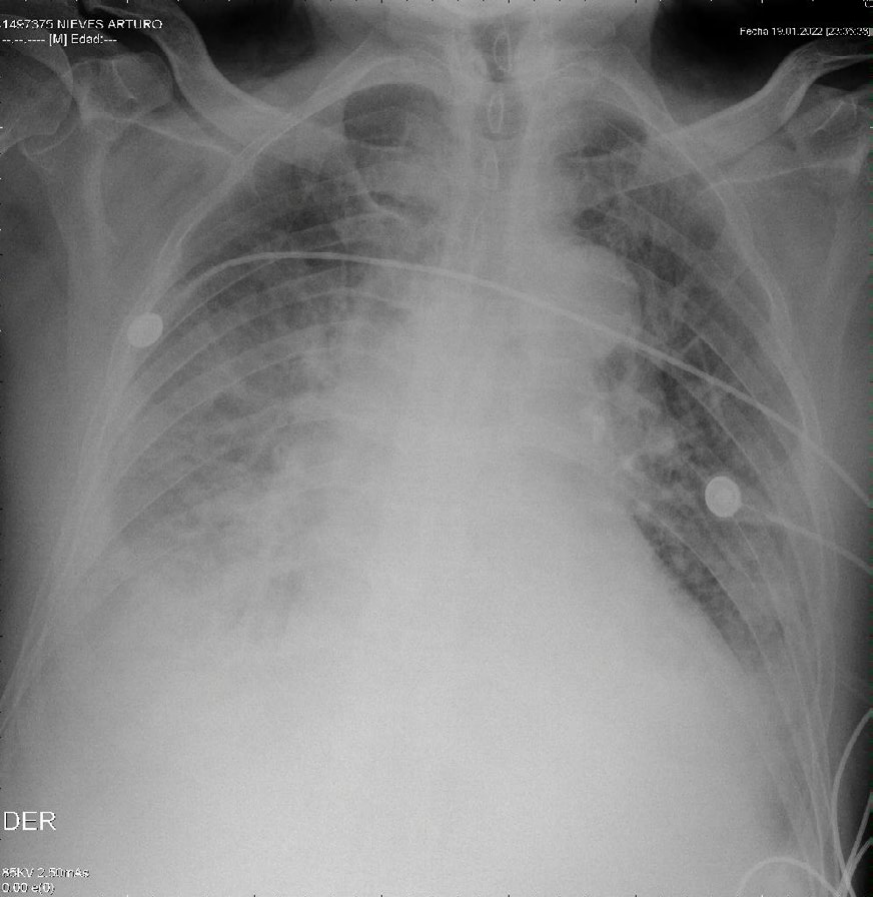
Chest X-ray upon admission (01/13/2023)

We present the case of an 83-year-old male patient with incomplete COVID-19 immunization schedule and no influenza vaccination. His medical history included heart failure, severe aortic insufficiency, arterial hypertension, and a history of smoking. The patient was admitted to the emergency department on 01/13/23 with clinical symptoms of 1 month of evolution, which consisted of asthenia, adynamia, paroxysmal nocturnal dyspnea, orthopnea, and edema in the lower limbs. A diagnosis of decompensated congestive heart failure with acute pulmonary edema was made (Figure 1). A transthoracic echocardiogram was performed on 01/21/2023 with a reported left ventricular ejection fraction of 55%, trivalve aortic valve with sclerosis, severe insufficiency, and 25 mm annulus and mitral valve with moderate to severe insufficiency. Significant obstructive coronary artery disease was ruled out by cardiac catheterization on 01/26/2023. Subsequently, the patient was evaluated at the cardiac surgical board where he was admitted to the protocol for transcatheter aortic valve implantation (TAVI).

**Figure 2:**
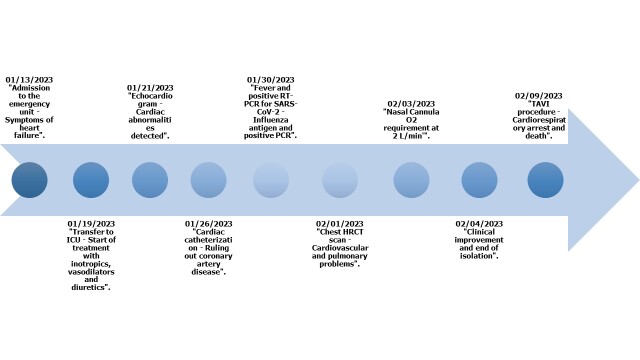
Timeline

*Note: RT-PCR: reverse transcription polymerase chain reaction, SARS CoV 2: severe acute respiratory syndrome coronavirus 2, PCR: polymerase chain reaction, O2: Oxygen, TAVI: transcatheter aortic valve implantation, ICU: intensive care unit, HRCT: high-resolution computed tomography. *

The patient was transferred to the intensive care unit (ICU) on 01/19/2023 and required use of inotropic, vasodilator and diuretic medications. After 7 days of clinical improvement, he was moved to the general ward. On 01/30/2023 he developed a fever without other associated symptoms. Laboratory findings revealed elevated inflammatory markers (C-reactive protein [CRP] 117.7 mg/L), lymphopenia (240 lymphocytes/µL) and increased D-dimer (21.11 µg/mL). A SARS-CoV-2 reverse transcription polymerase chain reaction (RT-PCR) test was ordered and returned positive (CT Gen N: 15). Given that more than 15 days had elapsed since initial emergency room admission until the onset of fever and symptoms compatible with COVID-19, so the possibility of nosocomial infection was raised. Among the variables associated with poor prognosis, the patient presented lymphopenia, increased D-dimer, elevated CRP, and advanced age. Due to clinical deterioration given by elevated respiratory rate (RR) (>24 breaths/minute), oxygen saturation (SaO₂) of 86% and moderate oxygenation disorder (PaO₂/FIO₂ 180), the patient required oxygen support with nasal cannula at 2 L/min, considering the initiation of intravenous steroid. Prior to its onset, studies were indicated to rule out influenza infection. The antigen and PCR report for influenza was positive, confirming coinfection with SARS- CoV-2 and influenza. Figure 2 illustrates the detailed timeline of key events that occurred during the patient’s hospital stay.

**Figure 3:**
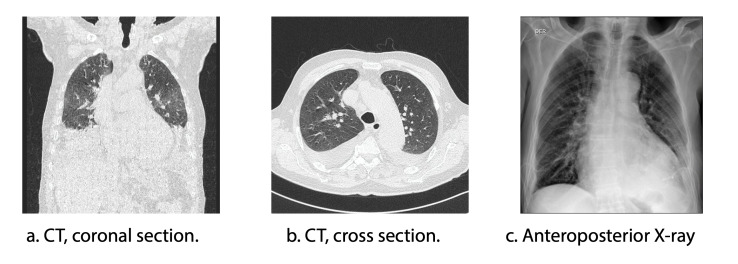
Chest X-rays

*Note: CT scan: computed axial tomography*

To assess the pulmonary parenchyma (Figures 3a and 3b), a high-resolution computed tomography scan of the chest (HRCT) was performed on 02/01/2023. The findings were consistent with dilated cardiomyopathy, precapillary pulmonary hypertension, coronary artery atherosclerosis, and large bilateral pleural effusions, most likely secondary to heart failure and fluid overload. No radiological evidence of pneumonia was observed. Multiple mediastinal lymph nodes of indeterminate etiology were identified. Due to pleural effusions, whose origin was considered to be heart failure, diagnostic and therapeutic thoracentesis were performed with transudate criteria (02/03/2023).

Management of the COVID-19 and influenza coinfection included oral oseltamivir at a dose of 75 mg every 12 hours for 5 days, in accordance with IDSA guidelines. Corticosteroid therapy was contraindicated due to the confirmed influenza infection. The patient showed progressive clinical improvement (SaO₂ 96%, RR 16 rpm, and FIO₂ 24%) (Figure 3c), showing a favorable clinical evolution. However, despite this improvement, the severe cardiovascular condition persisted and required intervention. The decision to undergo cardiovascular surgery was carefully discussed in a multidisciplinary team consisting of cardiology and infectious disease specialists. Risks and benefits were evaluated, considering the high probability of complications given the recent history of two severe infections. Finally, it was decided to perform the surgical procedure (08/09/2023). Unfortunately, the patient experienced cardiorespiratory arrest during the procedure and died.

A literature review was conducted in PubMed, Scopus, ScienceDirect, and Google Scholar databases, in Spanish and English, between January 2020 and June 2024. Although cases of coinfection with SARS-CoV-2 and influenza have previously been documented in Colombia, they remain scarce and are often limited in their clinical descriptions. This highlights the importance of reporting additional cases that provide detailed analyses of diagnostic and therapeutic approaches.

All procedures described in this case report were conducted in accordance with the ethical and bioethical standards established by the Scientific Committee of the treating medical institution, as well as with the 1964 Declaration of Helsinki and its subsequent amendments. Informed consent was obtained from the patient prior to participation and to the publication of this report and its accompanying images. The case report adheres to the international CAse REport guidelines. The data collected is fully available for free access and consultation in Mendeley Data^5^.

## Discussion

A novel coronavirus responsible for respiratory disease was first detected in December 2019 in Wuhan, China, and became a pandemic within a couple of months^1^,^5^. COVID-19 reached Latin America in February 2020, and was first detected in Colombia in March of that year^6^,^7^. The mortality rate for COVID-19 in Colombia is 3.8%, with arterial hypertension being the most common associated condition, although chronic obstructive pulmonary disease and hypothyroidism are also relevant^8^.

In addition to underlying medical conditions that increase morbidity and mortality in patients with COVID-19, coinfections have also been associated with worse clinical outcomes. The first reported case of SARS CoV-2 and rhinovirus/enterovirus coinfection developed severe multilobar pneumonia requiring ICU support^9^. Additionally, cases of coinfection by fungi and bacteria have been described^8^. However, COVID-19 mainly affects the lungs and can cause multiorgan failure and death similar to influenza^2^. Treatment guidelines for COVID-19 have proven effective in moderate to severe cases of the disease; however, they may be harmful in patients with influenza. This represents a challenge for healthcare professionals when evaluating both infections and seeking to reduce patient mortality.

The virus is transmitted through the respiratory tract, causing upper and lower respiratory symptoms and may progress to pneumonia^10^. Due to its pathophysiological characteristics, it has widespread distribution in the lungs, kidneys, gastrointestinal tract, and oral and nasal mucosa, triggering a systemic inflammatory response that can result in multisystemic damage. In severe cases, it requires ICU admission and is associated with high mortality due to multiorgan failure^10^.

One of the factors that affects the natural course of the disease in COVID-19 is coinfection with other respiratory pathogens that can complicate diagnosis, treatment and prognosis, generating a challenge when making clinical decisions^11^. In this context, the possibility of nosocomial infection should be considered, particularly in hospitalized patients who develop clinical symptoms a considerable time after admission—as was the case with our patient, who presented with fever and symptoms consistent with COVID-19 more than 15 days after his arrival at the emergency department. COVID-19 coinfection with bacteria and viruses has been documented at a rate of 7% and 3% respectively^6^. Coinfection by COVID-19 and influenza is rare. We are reporting the first clinical case of coinfection between COVID-19 and influenza in our country.

Screening studies report more cases, suggesting that unless patients with COVID-19 are actively screened, coinfection remains undiagnosed and underestimated and even more so depending on the screening method used. Therefore, it is important to consider the differential diagnoses at the time of patient examination. A meta-analysis indicated that 1.2% of patients with COVID-19 had an influenza coinfection^6^. In Colombia, a narrative review documented that dengue virus, Klebsiella pneumoniae, Mycobacterium tuberculosis, Pneumocystis jirovecii, Cryptococcus neoformans, and rhinovirus/enterovirus are among the most commonly reported coinfections with SARS-CoV-2^12^.

In Wuhan, China, an incidence of 0.8% was described^1^, while other studies describe an incidence between 0.24% and 44%^7^, which shows a high variability in the literature. Compared with the scientific literature, the proportion of influenza coinfection cases in our region appears to be lower.

In another study, Kim et al.^13^ evaluated 1217 patients with the following respiratory symptoms: 9.5% of the samples tested positive for SARS-CoV-2, and 26.1% were positive for other respiratory pathogens, including rhinovirus/enterovirus, respiratory syncytial virus, and other coronaviruses. Ding et al.^14^ included 115 patients with SARS-CoV-2 infection, 4.3% of which had coinfection with influenza. Additionally, Khodamoradi et al.^15^ reported other cases of patients with severe pneumonia due to SARS CoV-2 and influenza coinfection. This suggests that cases have been described worldwide, but few cases have been described in our population.

The differential diagnosis of both diseases is complex, since the clinical manifestation of both conditions is similar^16^,^17^. In patients with COVID-19, blood tests often reveal leukopenia and lymphopenia, while chest CT typically shows ground-glass opacities. Unfortunately, these findings are also observed in influenza A and other respiratory viral infections^18^. However, there are cases described with different radiological findings^19^. Radiology has a key role in clinical decision making in patients with suspected COVID-19^20^,^21^.

The importance of studying coinfection lies in the fact that both viruses are airborne pathogens affecting the respiratory tract. From a pathophysiological standpoint, coinfection with COVID-19 and influenza may worsen the patient’s prognosis due to the interaction of both viruses within the respiratory system. SARS-CoV-2 and influenza primarily target type II alveolar cells (AT2 pneumocytes), which can lead to significant lung damage, exacerbate the inflammatory response, and increase the risk of respiratory failure. Additionally, coinfection with these viruses is associated with a higher risk of complications, such as multiorgan failure, which increases mortality in this group of patients^7^. This would explain their similar pathophysiology and clinical presentation. It becomes a real diagnostic challenge to deal with patients with clinical symptoms and images highly suggestive of COVID-19, but with negative molecular samples.

Influenza can be diagnosed through viral culture, antigen detection, or nucleic acid testing. Viral culture is the most sensitive method, with sensitivity close to 100%; however, it requires 3–10 days to produce results. RT-PCR is considered the gold standard due to its high sensitivity. However, it is expensive and requires specialized personnel. Antigen detection using immunochromatographic assays provides results in a very short time (<30 minutes), but it is the least sensitive of the available methods^4^,^22^. This could explain the under-diagnosis of influenza when using the antigen as a diagnostic tool in our context. It should be noted that the diagnosis of influenza in our patient was initially using antigen and was confirmed through molecular testing. Prevalence studies have reported that coinfection with type A influenza virus is more common than with type B^23^, as observed in our case. No differences in mortality between influenza serotypes and COVID-19 have been reported.

Treatment of COVID-19 and influenza coinfection has been controversial. It has been described that the patients with COVID-19 managed with oseltamivir, an antiviral used in influenza infection had an increased risk of mortality^24^. Likewise, steroid therapy used in COVID-19 is contraindicated in influenza infection given the trend of increased mortality in this group^25^. However, an antiviral treatment with oseltamivir within the first 48 hours of COVID-19 and influenza coinfection has been reported to reduce disease burden, mortality, and hospitalization rates^26^. In our case, the use of steroids was contraindicated due to influenza infection and treatment with oseltamivir was indicated, with satisfactory clinical response.

According to the recommendation of the Colombian Association of Infectious Diseases, the waiting time for elective surgery after having had a SARS-CoV-2/COVID-19 coinfection should be 42 days given its high mortality risk. A publication by Wang et al.^17^ showed that the postsurgical complications in patients with COVID-19 with pulmonary involvement in the first 30 days was approximately 50%, and mortality could be as high as 38% compared to 24% in patients without pulmonary involvement^27^. It is important to note that the patients diagnosed with COVID-19 who are scheduled to undergo cardiac surgery face increased morbidity and mortality intraoperatively and postoperatively^13^,^28^. This is largely due to both their baseline cardiac condition and the state of systemic inflammatory response characteristic of virus infection, contributing to increased cardiometabolic demand due to lesions in the vascular endothelium and cardiac myocytes. Additionally, factors such as the need for mechanical ventilation and ischemia–reperfusion process also contribute to their increased morbidity and mortality^28^.

Despite recent severe infections, the decision to proceed with cardiovascular surgery was made after a thorough multidisciplinary assessment, which considered the severity of the patient’s underlying cardiovascular disease. Severe aortic valve insufficiency and congestive heart failure were an immediate threat to life, and surgical intervention was crucial to improve cardiovascular function and reduce the risk of long-term fatal complications. Choosing not to proceed with surgery would have carried a high risk of progressive deterioration in cardiac function, potentially leading to an even worse outcome. Although the patient’s recent infections and postinfectious phase increased the overall risk, the medical team decided to proceed with the intervention, carefully weighing the potential benefits of improving cardiovascular function against the inherent risks of coinfection and surgical complications. Unfortunately, the outcome was fatal, but the decision was based on the clinical judgment about the urgency of the cardiac condition and limited treatment options without surgery.

Risk factors such as age, gender, and the presence of comorbidities (such as hypertension, diabetes, and chronic respiratory diseases) are key determinants in the prognosis of coinfected patients. Studies indicate that the elderly individuals and individuals with underlying conditions are at increased risk of serious or fatal complications due to these coinfections^29^. In our population, mortality related to COVID-19 and influenza coinfection seems to be similar to that observed in other regions, although with some differences depending on access to medical treatment and the effectiveness of public health policies^29^.

In summary, limitations of this case report include the lack of a larger sample and variability in diagnostic methods used in the region. Furthermore, it is crucial to emphasize that, despite its limitations, this report contributes significantly to the understanding of coinfections in the local context, providing valuable information for clinical decision making and the design of future research. The strengths of this study include the early identification of coinfection in a critically ill patient and possibility of contributing to a better understanding of the factors that influence health outcomes in Colombia and Latin America.

## Conclusions

Although the two diseases share similar symptoms and clinical manifestations, their management and treatment can differ significantly. Accurate diagnosis is often complicated by overlapping clinical features and radiological findings. Coinfection, though uncommon, presents a considerable challenge for healthcare professionals due to its therapeutic implications. Radiological assessment and early detection play a critical role. Moreover, caution is warranted in postdiagnostic procedures, such as surgery, given the high risk of complications and associated mortality. A multidisciplinary approach is essential to optimize clinical outcomes in these patients, and early suspicion of coinfections is key to achieving timely diagnosis and appropriate treatment.
